# Population Impact & Efficiency of Benefit‐Targeted Versus Risk‐Targeted Statin Prescribing for Primary Prevention of Cardiovascular Disease

**DOI:** 10.1161/JAHA.116.004316

**Published:** 2017-02-10

**Authors:** Mark J. Pletcher, Michael Pignone, Jamie A. Jarmul, Andrew E. Moran, Eric Vittinghoff, Thomas Newman

**Affiliations:** ^1^ Department of Epidemiology and Biostatistics University of California, San Francisco San Francisco CA; ^2^ Department of Medicicine University of California, San Francisco San Francisco CA; ^3^ Department of Pediatrics University of California, San Francisco San Francisco CA; ^4^ Dell Medical School University of Texas at Austin TX; ^5^ Department of Health Policy and Management Gillings School of Public Health Chapel Hill NC; ^6^ School of Medicine University of North Carolina Chapel Hill NC; ^7^ Division of General Medicine Columbia University Medical Center New York NY

**Keywords:** cholesterol, population, risk assessment, statin, Epidemiology, Cardiovascular Disease, Primary Prevention, Lipids and Cholesterol

## Abstract

**Background:**

Benefit‐targeted statin prescribing may be superior to risk‐targeted statin prescribing (the current standard), but the impact and efficiency of this approach are unclear.

**Methods and Results:**

We analyzed the National Health and Nutrition Examination Survey (NHANES) using an open‐source model (the Prevention Impact and Efficiency Model) to compare targeting of statin therapy according to expected benefit (benefit‐targeted) versus baseline risk (risk‐targeted) in terms of projected population‐level impact and efficiency. Impact was defined as relative % reduction in atherosclerotic cardiovascular disease in the US population for the given strategy compared to current statin treatment patterns; and efficiency as the number needed to treat over 10 years (NNT_10_, average and maximum) to prevent each atherosclerotic cardiovascular disease event. Benefit‐targeted moderate‐intensity statin therapy at a treatment threshold of 2.3% expected 10‐year absolute risk reduction could produce a 5.7% impact (95% confidence interval, 4.8–6.7). This is approximately equivalent to the potential impact of risk‐targeted therapy at a treatment threshold of 5% 10‐year atherosclerotic cardiovascular disease risk (5.6% impact [4.7–6.6]). Whereas the estimated maximum NNT_10_ is much improved for benefit‐targeted versus risk‐targeted therapy at these equivalent‐impact thresholds (43.5 vs 180), the average NNT_10_ is nearly equivalent (24.2 vs 24.6). Reaching 10% impact (half the Healthy People 2020 impact objective, loosely defined) is theoretically possible with benefit‐targeted moderate‐intensity statins of persons with expected absolute risk reduction >2.3% if we expand age eligibility and account for treatment of all persons with diabetes mellitus or with low‐density lipoprotein >190 mg/dL (impact=12.4%; average NNT_10_=23.0).

**Conclusions:**

Benefit‐based targeting of statin therapy provides modest gains in efficiency over risk‐based prescribing and could theoretically help attain approximately half of the Healthy People 2020 impact goal with reasonable efficiency.

## Introduction

Statins are effective at reducing the incidence of cardiovascular disease events.^1^ Current guidelines recommend primary prevention with statins targeted according to a person's estimated 10‐year risk of atherosclerotic cardiovascular disease (ASCVD) events (myocardial infarction, fatal and nonfatal stroke, or coronary heart disease death) with a treatment threshold of 7.5%, such that statins are recommended for all persons with 10‐year risk >7.5%.^2^ Some have proposed targeting therapy instead according to the expected benefit from statins (measured by the absolute reduction in risk of events expected from statin therapy).^3^ Whereas baseline risk is a good marker for expected benefit, the expected benefit may also depend on individual characteristics that modify statin effectiveness (eg, a person with very low baseline lipid levels might have moderately high baseline ASCVD risk, but relatively low expected benefit from statins^1^). Benefit‐targeted primary prevention with statins may therefore be more efficient than risk‐targeted primary prevention.

It is unclear, however, how much a switch to benefit‐targeted statin therapy from risk‐targeted statin therapy would actually benefit the US population. A previous analysis projected that switching from risk‐targeted statin therapy with a baseline risk treatment threshold of 7.5% to benefit‐targeted statin therapy with an expected absolute risk reduction (ARR) treatment threshold of 2.3% could lead to treatment of an additional 9.5 million Americans and prevent an additional 266 508 cardiovascular events over 10 years^3^; but an equivalent impact could be obtained by simply lowering the baseline risk treatment threshold and treating more lower‐risk people. Lowering the treatment threshold would reduce prevention efficiency to some extent, but the degree is unclear. A direct comparison of the efficiency of benefit‐targeted versus risk‐targeted statin therapy at equivalent‐impact treatment thresholds has not yet been presented.

We used the open‐source Prevention Impact and Efficiency Model^4^ to illustrate and evaluate the impact‐efficiency trade‐offs of risk‐targeted versus benefit‐targeted statin therapy. We specifically evaluated efficiency of these strategies over a range of equivalent‐impact treatment thresholds, and compared the efficiency gains from benefit‐targeted statin therapy with gains that might be expected from improved risk prediction or from more‐efficacious prevention (ie, higher‐intensity statin therapy), and put results in the context of the Healthy People 2020 impact objective of reducing cardiovascular disease deaths by 20%.^5^

## Methods

### Model Overview and General Approach

We used the Prevention Impact and Efficiency (PIE) Model to compare statin prescribing targeted by baseline risk versus expected benefit. The PIE Model is a microsimulation modeling method and open‐source implementation, programmed in Stata, that is designed to illustrate trade‐offs between impact and efficiency of targeted prevention interventions.^4^ The PIE Model estimates impact (in terms of relative % reduction in disease events) and efficiency (in terms of the number of persons needed to treat [NNT] to prevent each event) by (1) estimating baseline risk for each National Health and Nutrition Examination Survey (NHANES) participant (using measurements from NHANES and applying an externally derived risk prediction algorithm); (2) estimating the reduction in disease risk in each NHANES participant that might be achieved from an intervention, and applying it to participants who meet targeting criteria; (3) estimating overall population average risk before and after targeted treatment with the intervention to estimate relative % reduction in disease (impact); and (4) estimating the average and maximum NNT to prevent each disease event (efficiency). We used PIE Model methods to analyze NHANES, a nationally representative cross‐sectional survey of the US population, to produce estimates of impact and efficiency of benefit‐targeted and risk‐targeted statin therapy. The model implementation files used for this analysis, programmed in Stata (version 13; StataCorp LP, College Station, TX), are archived and publicly available on the website.^4^ Besides the simple example on the GitHub website (“PIEModelTemplate_v1.0”), this analysis is the first published use of the PIE Model.

### Target Population and Study Sample

We defined our target population as US adults, and our study sample as participants in the NHANES 2011–2012 fasting laboratory testing subsample who were at least 20 years of age. For our primary analysis, we used multiple imputation (n=10 imputed data sets generated) to impute missing values of key variables (2.6% of all required measurements are imputed—see Table S1) so that all adults participating in the NHANES fasting laboratory testing sample were included and our estimates are generalizable to the US population. Sampling weights provided with NHANES for use with the fasting laboratory testing subsample were used in all analyses. NHANES is publicly available, and our analysis is exempt from institutional review board review.

### Estimating Baseline 10‐Year Risk of Atherosclerotic Cardiovascular Disease

Our target condition for this analysis is cardiovascular disease events. To estimate baseline risk of cardiovascular disease events in all adult patients in the population (required for calculation of population impact in terms of relative % reduction in events), we combined information from several published cardiovascular risk equations and used additional assumptions regarding risk and intervention efficacy in statin users.

For persons without pre‐existing cardiovascular disease, we used the 2013 American College of Cardiology/American Heart Association Guideline on the Assessment of Cardiovascular Risk^2^ to estimate 10‐year risk of ASCVD, defined as fatal and nonfatal myocardial infarction and stroke, for black and white men and women using age, sex, systolic blood pressure, total and high‐density lipoprotein (HDL) cholesterol, blood pressure medication use, current smoking status, and diabetes mellitus measurements. To estimate risk for NHANES participants who were Hispanic, Asian, or another race/ethnicity besides black or white, we used the parameters designed for white men and women, as suggested by the Guideline.^2^

For persons reporting pre‐existing cardiovascular disease (“Has a doctor or other health professional ever told you that you had” coronary heart disease, angina, heart attack, or stroke), we used a Framingham Heart Study–based calculator designed for calculation of 2‐year risk of recurrent coronary heart disease (myocardial infarction, coronary insufficiency, angina pectoris, and sudden and nonsudden coronary death; stroke is not included).^6^, ^7^ We calculated 2‐year risk with current age and current risk factor levels and then recalculated risk every 2 years (assuming that age increases, but no other risk factors change) in order to extrapolate risk to 10 years, and then assumed that this estimate was comparable to the ASCVD risk calculator described above. This imperfect assumption allows us to make population‐level impact estimates in terms of relative % reduction in events in the US population.

These risk estimates assume no treatment with statin therapy. In order to estimate baseline risk in a statin user, we estimated pretreatment lipid levels, calculated risk estimates (as above) using these estimated pretreatment values, and then applied statin‐associated relative risk reductions to these pretreatment risk levels.^1^ For persons reporting current use of Pravastatin, Lovastatin, Simvastatin, or Fluvastatin, we estimated pretreatment lipid measurements assuming these medications had effects on total cholesterol (27% reduction), low‐density lipoprotein (LDL) cholesterol (34% reduction) and HDL cholesterol (5% increase)^8^; for persons reporting current use of Atorvastatin, Rosuvastatin, or Pitavastatin, we assumed these medications had larger effects on total cholesterol (37% reduction), LDL cholesterol (48% reduction), and HDL cholesterol (5% increase)^8^; see Table S2 for tabulation of statin users in the NHANES target population.

### Estimating Reduced Cardiovascular Risk With Moderate‐Intensity Statins

We modeled statin effectiveness using methods and assumptions developed by Thanassoulis et al.^3^ We defined moderate‐intensity statins as statin therapy that achieves an LDL reduction of 40% in persons who were not already taking a statin. We translated the effects of LDL‐lowering from statins into a reduced event risk by applying the ARR formulation developed by Thanassoulis et al,^3^ which is derived from the Cholesterol Treatment Trialists (CTT) meta‐analysis that demonstrated an interaction in statin efficacy by baseline level of risk (formulas provided by Thanassoulis et al in their online appendix^1^). We used this calculated value—the expected ARR—both to estimate the reduction in risk from statins for each NHANES participant treated with statins (for both benefit‐ and risk‐targeted therapy) as well as to target that therapy (for benefit‐targeted therapy only). The PIE Model Stata code implementing calculation of the expected ARR is archived and available on the website^4^; the critical steps are described below:

1. lnRRthanassoulis=−0.10821+0.12346*ln(baselinerisk) (from Thanassoulis et al^3^)
2. RRthanassoulis=exp(lnRRthanassoulis) (RR *per mmol/L LDL reduction*)
3. RR=RRthanassoulis^(LDLreduction in mmol/L) (Calculated RR for given patient)

To focus our base‐case analysis on comparison of risk‐targeting versus benefit‐targeting of statins for primary prevention, we did not apply statin therapy in persons with previous cardiovascular disease or diabetes, or with LDL ≥190 mg/dL, because statins are indicated in these groups regardless of risk or expected statin benefit.^2^ These persons are effectively omitted from our analysis, except that they figure into the overall impact calculation given that they are still part of the US population.

### Estimating Impact and Efficiency for Each Treatment Threshold

We serially analyzed prevention impact and efficiency for a range of potential thresholds in baseline risk, and in expected ARR, above which statins might rationally be applied. To estimate prevention impact (relative % reduction in events in the population), we compared pre‐ to post‐intervention population‐averaged cardiovascular risk (calculated using study weights) (=1−(Average_Risk_post‐intervention_/Average_Risk_pre‐intervention_), then multiplied by 100 and expressed as a %). To estimate prevention efficiency, we estimated average and maximum NNT over 10 years (NNT_10_) to match the time frame of our risk estimates. To obtain an *average* NNT_10_ estimate, we calculated the mean risk difference (Risk_untreated_−Risk_treated_) among individuals in the treated subset (using study weights) for each strategy and then took the inverse of that mean. To obtain a maximum NNT_10_, we identified the single treated individual with the smallest risk difference and took the inverse of that value.^3^ These calculations are standard for the PIE Model.^4^

Although we focus analyses on point estimates, we also report 95% CIs for key estimates to illustrate imprecision resulting from both sampling error and multiple imputation. We calculate point estimates by analyzing all 10 imputed data sets together in 1 combined data set; the resulting combined estimates are asymptotically equivalent to estimates obtained from analyzing each imputed data set separately and then averaging the results (the standard method of analyzing multiple imputation data sets). To estimate CIs, we devised a novel Monte Carlo simulation approach. We first looped through each of the 10 imputed data sets individually, obtaining 1000 bootstrap samples^9^ from each imputed data set by resampling NHANES primary sampling units within strata. We then obtained key estimates in each simulated data set (n=10×1000=10 000 total runs per estimate) and reported the 2.5th and 97.5th percentile values as the 95% CIs for each result. This approach is necessary because, although Stata supports multiple imputation, bootstrapping, and complex survey data analysis, it does not support these methods in combination. Note that this Monte Carlo simulation method estimates error from sampling and imputation, but it does not account for uncertainty in our assumptions. We do not present CIs for maximum NNT_10_ because this value is entirely dependent (defined by) a single outlier value in the data set and does not represent a statistical estimate.

### Comparing Strategies

Our primary comparison was between risk‐targeted prescribing, defined as moderate‐intensity statins prescribed according to level of baseline risk, and benefit‐targeted prescribing, defined as moderate‐intensity statins prescribed according to level of expected ARR. Prevention impact‐efficiency curves are provided to illustrated the trade‐offs, labeling thresholds of 7.5% for baseline risk (the 2013 Guideline^2^), 2.3% for expected ARR (for comparison with previous analyses^3^), and equivalent‐impact thresholds.

**Figure 1:**
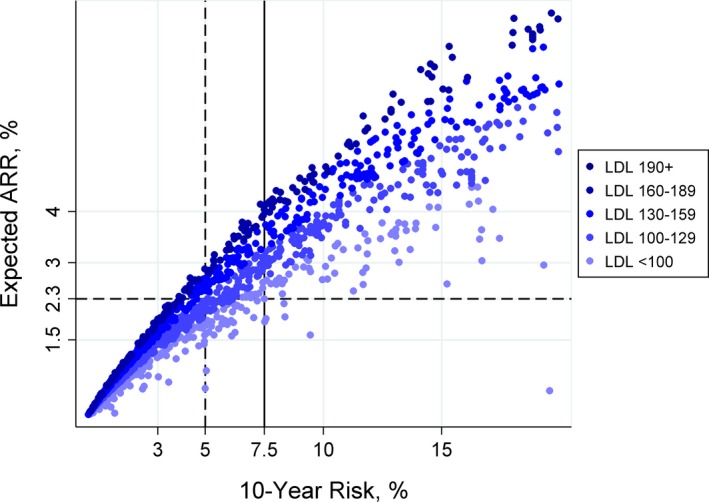
Estimated 10‐year atherosclerotic cardiovascular disease (ASCVD) baseline risk and expected absolute risk reduction (ARR) with moderate‐intensity statins. Expected ARR is correlated with baseline low‐density lipoprotein (LDL; *r*=0.34) and baseline risk (*r*=0.93). The solid reference line indicates the current standard treatment threshold (baseline risk >7.5%); the dashed reference lines indicate a proposed alternative (expected ARR >2.3%) and a risk‐targeted treatment threshold with approximately equivalent impact (baseline risk >5%, see Figure 2).

For comparison and context, we present results relative to a 10% impact goal, which is half of the Healthy People 2020 impact objective of reducing coronary heart disease deaths by 20%^5^ (loosely defined), assuming the other half should be attained by smoking cessation, lifestyle modification, blood pressure control, or other methods. We also present results for: (1) a hypothetical perfect risk prediction algorithm (where risk is 100% in the high‐risk group and 0% in the low‐risk group, and there is only 1 logical treatment threshold); (2) age‐targeted prescribing (a less‐accurate, but more easily applied method of statin targeting); (3) benefit‐targeted prescribing using the average relative risk reduction across baseline risk groups (0.75 per mmol/L LDL reduction for primary prevention and 0.80 for secondary prevention; see Figure 1 of published meta‐analysis^1^) without the baseline risk interaction modeled by Thanassoulis et al^3^; (4) high‐intensity statins (with 50% LDL reduction for comparability^3^); and (5) overall impact and efficiency accounting for treatment of all patients with diabetes mellitus, and LDL ≥190 mg/dL, and with expanded age eligibility for targeted statin therapy to include adults <40 and >75 years of age.

## Results

NHANES 2011–2012 includes 9756 individual participants, of whom 3239 participated in the fasting laboratory test subsample; 2627 were adults aged ≥20 years and represent our target population. Most participants had a complete set of measurements for our analysis (n=2290). Participants who were missing 1 or more required measurements had higher systolic blood pressure (*P*=0.023), higher total cholesterol (*P*=0.002), lower HDL (*P*=0.001), and were more likely to be diabetic (*P*=0.001; Table S1). With multiple imputation (creating a 10‐fold imputed data set with total N=10×2627=26 270; see Methods), we identified 7931 observations of persons eligible for targeted primary prevention with statin therapy (ie, aged 40–75, no previous cardiovascular disease or diabetes mellitus, not already on a statin, and with LDL <190 mg/dL), representing 34% of the US population (applying NHANES sample weights).

Among US adults eligible for targeted primary prevention with statins (Table 1), Expected ARR was correlated with LDL (*r*=0.34) and strongly correlated with baseline risk (*r*=0.93; Figure 1). We estimate that 4% of the US adult population (11% of the potentially eligible US population=8.4 million persons) would be reclassified from statin not indicated to indicated (expected ARR >2.3%) by switching from risk‐targeted prescribing at Guideline‐suggested threshold (baseline risk >7.5%^2^) to benefit‐targeted prescribing at the recently suggested expected ARR threshold (expected ARR >2.3%^3^). Only 0.03% of the US adult population (0.1% of the potentially eligible US population=30 observations in the 10‐fold multiple imputation data set=3 NHANES participants) were reclassified from statin indicated to not indicated (Figure 1; Table 1).

**Table 1:** Characteristics of US Adults Aged 40 to 75 and Eligible for Targeted Primary Prevention With Statin Therapy^a^, by Baseline 10‐Year ASCVD Risk and Expected ARR From Moderate‐Intensity Statins

Characteristic	Baseline Risk^b^ <7.5%		Baseline Risk^b^ >7.5%	
	Expected ARR^b^ <2.3%	Expected ARR^b^ >2.3%	Expected ARR^b^ <2.3%	Expected ARR^b^ >2.3%
N in US population^c^, in millions	49.3	8.4	0.07	17.8
% of US population^d^	22	4	0.03	8
Baseline risk^b^, %				
Min to Max	0.06 to 7.2	3.9 to 7.5	7.5 to 8.4	7.5 to 35
Median (interquartile range)	1.5 (0.9–2.9)	6.2 (5.1–6.9)	8.4 (7.5–8.4)	11 (9.4–16)
Expected ARR, %				
Min to Max	0.5 to 2.3	2.3 to 4.1	1.85 to 1.95	2.3 to 9.2
Median (interquartile range)	0.8 (0.5–1.4)	2.7 (2.5–3.1)	1.9 (1.85–1.95)	4.4 (3.7–5.3)
Age, mean years±SD	48±6	56±7	57±2	62±8
Sex, % male	35	45	100	72
Systolic blood pressure^b^, mean mm Hg±SD	118±13	127±21	126±6	131±17
Total cholesterol, mean mg/dL±SD	200±34	223±32	139±6	213±31
LDL cholesterol, current^a^, mean mg/dL±SD	120±30	141±24	64±1.0	130±28
HDL cholesterol, mean mg/dL±SD	56±15	54±16	54±12	52±16
Smoking, % current	12	34	0	31
Current blood pressure medication use, %	17	24	61	49
Diabetes mellitus^a^, %	0	0	0	0
Current statin use^a^, %	0	0	0	0
Prevalent ASCVD^a^, %	0	0	0	0

ARR indicates absolute risk reduction; ASCVD, atherosclerotic cardiovascular disease; HDL, high‐density lipoprotein; LDL, low‐density lipoprotein; Max, maximum; Min, minimum; NHANES, National Health and Nutrition Examination Survey; Statin, HMG Co‐A reductase inhibitor.

a NHANES participants younger than 40 years, older than 75 years, with LDL ≥190 mg/dL, with diabetes mellitus, with previous cardiovascular disease, or already on a statin are excluded from this analysis.

b Baseline risk is defined as 10‐year risk of ASCVD, estimated according to the 2013 Guideline^2^; expected ARR is the expected absolute risk reduction from moderate‐intensity statin therapy, as formulated by Thanassoulis et al^3^ see Methods.

c US population estimates are calculated from the Ns in NHANES. Ns for columns 1 to 4 in the 10‐fold imputed data set were 4725, 879, 30, and 2297, respectively; these Ns were divided by 10 to correct for the 10‐fold imputation and then multiplied by the sample weights provided by NHANES.

d US population % estimates use US population estimates^‡^ as the numerator, and all US adults in the denominator, such that the total % adds up to 34% (total % eligible for targeted primary prevention*) rather than 100%. Note: All subsequent results describe only eligible persons* and use this as the denominator (so that %s add up to 100%) and are also weighted using sample weights.

Compared with current treatment patterns, we estimate that benefit‐targeted prescribing of moderate‐intensity statins at an expected ARR threshold of 2.3% would reduce ASCVD events in the US population by 5.7% (95% CI, 4.8–6.7%), at an average NNT_10_ of 24.2 (23.1–25.4). In contrast, the impact of risk‐targeted prescribing of moderate‐intensity statins at a baseline risk threshold of 7.5% in the same population is smaller—4.4% (3.7–5.2%)—but the average NNT_10_ is also lower—21.2 (20.4–22.0), indicating better average prevention efficiency (Table 2).

**Table 2:** Impact and Efficiency Estimates for Risk‐Targeted Versus Benefit‐Targeted Prescribing of Moderate‐Intensity Statins

Targeting Strategy Treatment Threshold	Impact	Efficiency	
	Proportion of ASCVD Events Preventable % (95% CI^a^)	Average NNT_10_ (95% CI^a^)	Maximum NNT_10_ b
Risk‐based prescribing			
Treat all	8.3 (7.2–9.5)	48.3 (45.1–52.0)	2100
Treat if baseline risk^c^ >3%	6.8 (5.7–7.9)	29.1 (27.9–30.3)	180
Treat if baseline risk >5%	5.6 (4.7–6.6)	24.6 (23.5–25.8)	180
Treat if baseline risk >7.5%	4.4 (3.7–5.2)	21.2 (20.4–22.0)	54
Treat if baseline risk >10%	3.2 (2.6–3.7)	19.1 (18.3–19.9)	39
Treat if baseline risk >15%	1.6 (1.2–2.0)	16.2 (15.4–16.9)	39
Benefit‐based prescribing			
Treat all	8.3 (7.2–9.5)	48.3 (45.1–52.0)	2100
Treat if expected ARR^c^ >1.0%	7.5 (6.4–8.7)	33.6 (31.9–35.4)	100
Treat if expected ARR >1.5%	6.9 (5.8–8.0)	29.2 (27.8–30.7)	66.5
Treat if expected ARR >2.3%	5.7 (4.8–6.7)	24.2 (23.1–25.4)	43.5
Treat if expected ARR >3.0%	4.8 (4.0–5.7)	21.6 (21.0–22.2)	33.3
Treat if expected ARR >4.0%	3.3 (2.7–4.0)	18.6 (18.0–19.1)	24.9
Treat if expected ARR >5.0%	1.9 (1.5–2.3)	15.6 (15.0–16.2)	19.9

ASCVD indicates atherosclerotic cardiovascular disease; CIs, confidence intervals; NNT_10_, number need to treat over 10 years to prevent 1 event.

a CIs presented here are the 2.5th and 97.5th percentiles of the distribution of estimates derived from analyzing bootstrapped samples accounting for the complex National Health and Nutrition Examination Survey survey design and multiple imputation procedure.

b We do not present confidence intervals for maximum NNT_10_ because this value is entirely dependent (defined by) a single outlier value in the data set and does not represent a statistical estimate.

c Atherosclerotic cardiovascular disease (ASCVD) risk was estimated using the algorithm described in the 2013 American College of Cardiology/American Heart Association Guideline on the Assessment of Cardiovascular Risk^2^ for persons without pre‐existing cardiovascular disease, or an alternate Framingham‐based risk equation^6^, ^7^ with extrapolation to 10 years, for persons with and without pre‐existing cardiovascular disease, respectively.

**Figure 2:**
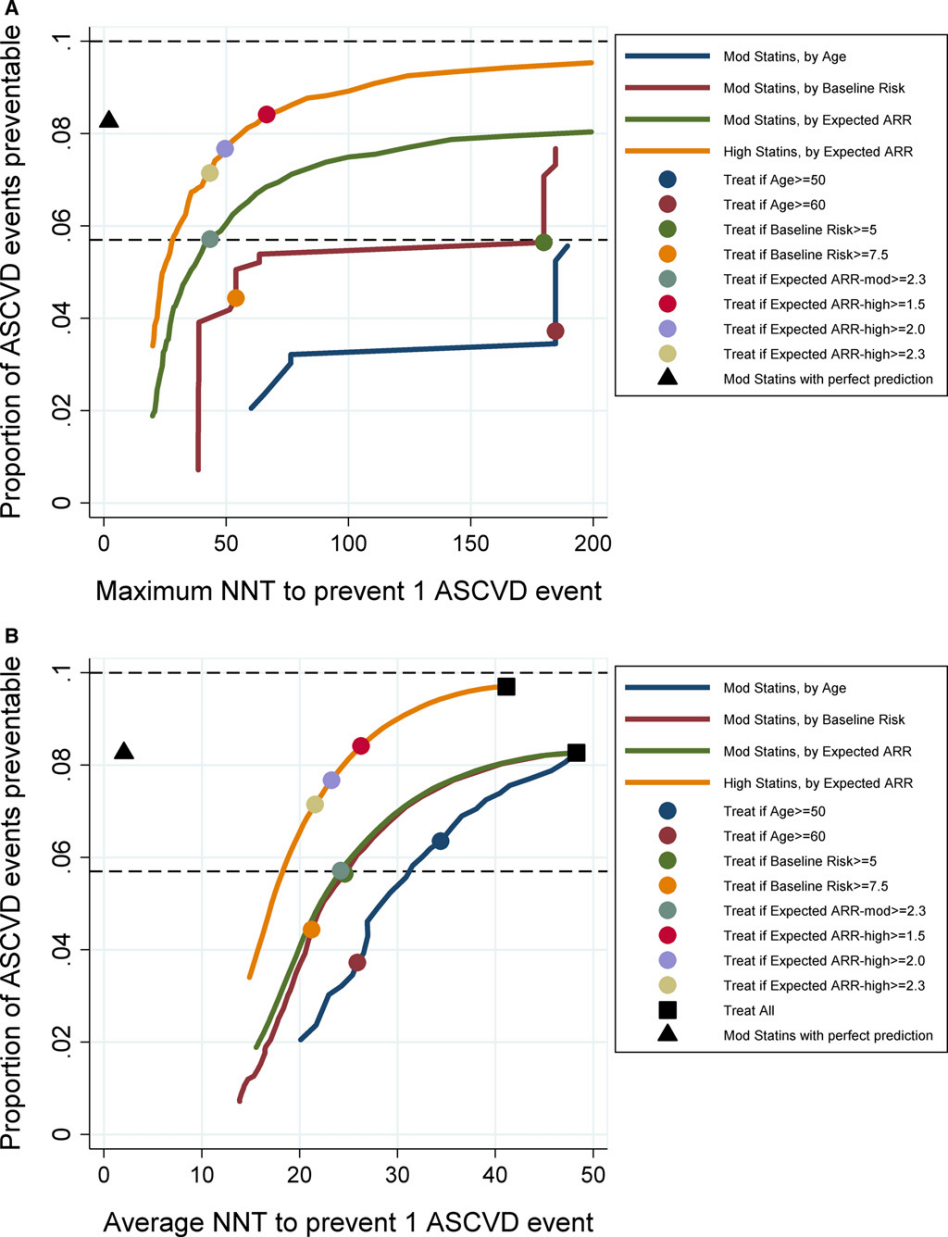
Prevention impact and efficiency of benefit‐ versus risk‐targeted statin prescribing. Impact (relative % reduction in ASCVD events) and number needed to treat (NNT) over 10 years to prevent each ASCVD event (lower NNT is more efficient) are illustrated across treatment thresholds for moderate‐ and high‐intensity statins targeted by expected absolute risk reduction (ARR) (benefit‐based targeting) and targeted by age or by baseline risk (risk‐based targeting) or by perfect prediction (theoretical only). Efficiency is plotted for both the maximum NNT (A) and average NNT (B). Dashed lines indicate 5.7% impact (equivalent to the impact of moderate‐intensity statins at expected ARR >2.3%) and 10% impact (a potential impact goal for statin prescribing that might help attain Healthy People 2020 objectives^5^). ASCVD indicates atherosclerotic cardiovascular disease.

A more‐direct comparison of prevention efficiency for different targeting strategies can be attained by choosing equivalent‐impact thresholds. To match the impact of benefit‐targeting at a threshold of 2.3%, we can lower the risk‐targeting threshold to ≈5% (Figure 2), which would produce 5.6% impact (4.7–6.6%). At this threshold, the average NNT_10_ is nearly equivalent—24.6 (23.5–25.8; compare with 24.2 as above)—though the maximum NNT_10_ becomes much higher—180 (compare with 43.5; Table 2).

The impact‐efficiency trade‐off for these strategies can be illustrated by plotting impact versus NNT_10_ (Figure 2). For any given level of impact, the maximum NNT_10_ is substantially lower for benefit‐targeted prescribing than risk‐targeted prescribing (in Figure 2A, the green curve is shifted toward the upper left corner compared with the red curve). The average NNT_10_, however, is nearly identical (Figure 2B). For comparison, we have plotted age‐targeted prescribing of moderate‐intensity statins, which is substantially *less* efficient by any metric; and benefit‐targeted high‐intensity statins, which is substantially *more* efficient and also potentially more impactful. For example, we project that high‐intensity statins prescribed for all persons with an expected ARR ≥2.3 would produce a 7.1% (6.0–8.4) impact at an average NNT_10_ of 21.6 (20.7–22.6) and a maximum NNT_10_ of 43.3. Although improved risk prediction will improve efficiency of targeted therapy, it will not improve potential maximum impact for any given strategy; for example, moderate‐intensity statins with even theoretical perfect prediction does not match the potential impact of high‐intensity statins prescribed for persons with expected ARR ≥1.5 (expected impact=8.4% [7.1–9.8] and average NNT_10_=26.3 [24.9–27.8]; Figure 2).

If our goal were to reduce cardiovascular disease by 10% with statins alone (half of the Healthy People 2020 impact objective of reducing coronary heart disease deaths by 20%, loosely defined), targeted statin prescribing as defined here would fall short (Figure 2). If we wanted to maximize impact, but keep maximum NNT_10_ <50 (equivalent to targeting at expected ARR >2.0%), we could achieve impact of 7.7% with high‐intensity statins (Figure 2). Even a treat‐all approach with high‐intensity statins (which has a very high NNT_10_—average=41.1, maximum [max]=1900) would achieve an impact of only 9.7%. However, if we additionally account for treatment of all persons with diabetes mellitus and LDL >190 mg/dL, and allow treatment of persons of any age with expected ARR >2.3, we could theoretically achieve an impact of 12.4% with benefit‐targeted moderate‐intensity statins or 15.3% with high‐intensity statins (Figure S1).

Sensitivity analyses demonstrate critical dependence of our absolute impact and efficiency estimates on the assumption that statins are relatively more effective (eg, the relative risk reduction from statins, per mg/dL reduction in LDL, is larger) in patients with lower baseline risk^1^ (see Methods). When we substitute the average relative risk per mmol/L reduction in LDL for patients without vascular disease reported by the CTT,^1^ primary prevention with statins (either risk‐ or benefit‐targeted) at any given threshold is substantially less impactful and less efficient, and the impact of 2.3% expected ARR treatment threshold is more akin to a baseline risk treatment threshold of 7.5% than 5% (Figure S2). Note that our finding of nearly identical average NNT for risk‐ versus benefit‐targeted statin therapy, however, is robust.

## Discussion

Risk‐targeted statin therapy leads to treatment of some individuals with a relatively low expected benefit from therapy (attributed to low baseline LDL levels), and treatment of these individuals is inefficient (ie, the maximum NNT_10_ is high). Benefit‐targeted statin therapy, in contrast, intrinsically maximizes efficiency and is a more‐rational choice for guiding clinical decisions. A switch to benefit‐targeted statin therapy, however, will provide only very small gains in average prevention efficiency compared with risk‐targeted statin therapy at equivalent‐impact treatment thresholds. More‐sizable gains in efficiency and/or impact are attainable with improvements in risk prediction or with more efficacious therapy (eg, high‐intensity statins). A 10% impact (half of the Healthy People 2020 impact objective, loosely interpreted) is not achievable with targeted statins alone, but could theoretically be achievable with expanded eligibility criteria and accounting for treatment of all persons with diabetes mellitus or LDL >190 mg/dL.

Our results are consistent with previous analyses by Thanassoulis et al,^3^ who proposed the benefit‐targeting approach, developed an expected ARR estimation algorithm^3^ using the interaction with baseline risk identified by the CTT,^1^ and estimated population‐level impact of using benefit‐targeted moderate‐intensity statins with an expected ARR treatment threshold of 2.3% by analyzing NHANES. Our analysis of NHANES, using identical assumptions and a similar approach, augmented by multiple imputation for enhanced generalizability to the US population, produced consistent estimates. We extended these findings by providing CIs estimated using Monte Carlo simulation that account for multiple imputation and sampling error in NHANES, illustrating the impact‐efficiency trade‐offs at different treatment thresholds; providing estimates of efficiency for equivalent‐impact treatment thresholds allowing for a more‐direct comparison of benefit‐ versus, risk‐targeted therapy; demonstrating theoretical impact in the context of the Healthy People 2020 impact objectives; and providing open‐source code for our model. We invite readers with access to Stata to download and modify model files to further explore these issues^4^; a sample of the Stata code required to produce prevention impact‐efficiency trade‐off curves for Figure 2A and Figure S1 is provided in Data S1.

We found that the assumption made by Thanassoulis et al^3^ of an interaction between baseline risk and statin effectiveness has important implications for estimating impact and efficiency of statin therapy, and also for how to implement benefit‐targeted statin therapy. Unlike risk‐based targeting, benefit‐based targeting would use estimated expected ARR for clinical decision making about statin treatment for individual patients, and the expected ARR is very sensitive to the interaction assumption. Thanassoulis et al^3^ did not evaluate impact with a no‐interaction assumption. The interaction assumption is justified by the presence of a statistically significant interaction detected by the CTT^1^ (*P*=0.003; see Figure 2, 1); however, that analysis starts with an assumption that the effectiveness of statins scales with the degree of LDL reduction^1^ (the relative risk from statin use is provided per 1 mmol/L LDL reduction), which is, in turn, dependent on the baseline LDL level.^8^ This assumption has been controversial, with some arguing that the effectiveness of statins varies only by statin characteristics (potency) and not patient characteristics (baseline LDL level),^10^ a position supported by recent evidence.^11^ Indeed, current guidelines do not recommend consideration of baseline LDL level in statin decision making unless it is extremely high (>190 mg/dL).^1^ Our analysis demonstrates the urgency of resolving this fundamental question about statin effectiveness, especially if a benefit‐based approach to statin prescribing is to be implemented.

Our approach has some limitations. Our estimates assume perfect implementation by clinicians and perfect acceptance and adherence by patients and are thus only theoretically attainable. The estimates are only as good as our assumptions (eg, risk prediction and statin effectiveness estimates), and our 95% CIs account only for random sampling and imputation error and not for uncertainty in the assumptions themselves (eg, statin efficacy); we have not attempted to evaluate these sources of uncertainty, and our projections should be considered dependent on the accuracy of these assumptions. Alternate assumptions would be easy to evaluate by modifying the open‐source implementation files archived on the PIE Model website.^4^ Unlike Thanassoulis et al,^3^ we have not attempted to evaluate or incorporate a trials‐based approach to selecting patients and targeting statin therapy, and we have made the assumption that statin effectiveness (modeled by the algorithm proposed by Thanassoulis et al^3^) is uniform across the population and independent of medical condition, age, C‐reactive protein levels,^12^ or other factors. The 10% impact goal we use as a benchmark in our analyses is relevant, but not equivalent, to (half of) the Healthy People 2020 objective of reducing coronary heart disease deaths by 20%,^5^ given that deaths may be reduced by a different proportion than total ASCVD events. Our modeling approach uses a simple fixed horizon at 10 years and does not attempt to model downstream events after the occurrence of an ASCVD event (or even to distinguish fatal from nonfatal ASCVD events). Also, to obtain population‐level impact estimates, we needed to combine risk estimates from an ASCVD risk calculator and a recurrent coronary heart disease calculator, and make additional assumptions about detreating/retreating with statins. We do not attempt to model adverse effects from statins or estimate cost or cost‐effectiveness. Finally, note that our impact and NNT estimates are only relevant to the proposed use of statins under a given policy (ie, treatment threshold) in comparison to current treatment patterns; that is, the impact and efficiency of prevalent statin use is used to define the baseline scenario, excluded from the proposed intervention (because it has already occurred), but accounted for when estimating impact and efficiency of a proposed alternative scenario (because prevalent statin users are still part of the US population “denominator” for relative % reduction impact estimates).

Although benefit targeting of statin therapy produces only very small average gains in efficiency compared to risk‐targeting at equivalent‐impact treatment thresholds, benefit‐based targeting does limit treatment of some individuals with a small expected benefit and is, in this sense, a more rational approach to targeted prevention. In fact, it is the theoretical ideal for maximizing targeted treatment efficiency for any given targeted prevention therapy and provides an excellent framework for rational translation of precision medicine discoveries into gains in public health. Microsimulation methods are naturally suited to modeling impact and efficiency of benefit‐targeted precision medicine, and the PIE Model, which uses these methods, may be generally useful for helping translate precision medicine interventions into guidelines and policy. For statin therapy, the impact of benefit‐targeted therapy compared to risk‐targeted therapy is small; it is also highly dependent on our assumptions about how we model statin efficacy, as described above. But benefit targeting can help us apply evidence, when available, of heterogeneity in statin effectiveness (eg, from biomarkers like C‐reactive protein^12^ or genetic testing^13^) that precision medicine methods will help to generate. Before we can reap these benefits, however, we will need more consensus and updated guidelines that sanction this approach, as well as clinician‐friendly tools to calculate expected benefit similar to what is now available for calculating baseline risk.^14^

## Sources of Funding

This work was supported by the National Institutes of Health Economics of Prevention Common Fund Initiative, through a grant from the National Heart, Lung, and Blood Institute (R21HL112256).

## Disclosures

None.

## Supporting information


**Data S1.** Two snippets of the PIE Model configuration code used to define Figure 2A and Figure S1.
**Table S1.** Characteristics of NHANES 2011–2012 Participants Aged ≥20 Years Using Sample Weights, With and Without Multiple Imputation
**Table S2.** Assumed Effects of Different Statins on Total, Low‐Density Lipoprotein (LDL), and High‐Density Lipoprotein (HDL) Cholesterol
**Figure S1.** Prevention impact and efficiency for moderate‐intensity and high‐intensity statins with and without expanded eligibility. Impact (relative % reduction in ASCVD events) and number‐needed‐to‐treat (NNT) over 10 years to prevent each ASCVD event (lower NNT is more efficient) are illustrated across treatment thresholds for moderate‐ and high‐intensity statins targeted by expected absolute risk reduction (benefit‐based prescribing) and either limited to persons age 40 to 75 years, LDL <190 mg/dL and without diabetes mellitus or previous cardiovascular disease (base‐case analysis); or with expanded eligibility accounting for treatment of all persons with diabetes mellitus and LDL ≥190 mg/dL, and treatment of all adults of any age meeting the given expected absolute risk reduction threshold. Dashed line indicates 10% impact. ASCVD indicates atherosclerotic cardiovascular disease; LDL, low‐density lipoprotein cholesterol.
**Figure S2.** Prevention impact and efficiency for moderate‐intensity statins with and without baseline risk interaction assumption. Our base‐case analyses assume an interaction between baseline risk and statin effectiveness, as detected by the Cholesterol Treatment Trialists (CTT)^1^ and operationalized by Thansssoulis et al^2^ (see Methods). This figure demonstrates that impact and efficiency are both substantially less favorable for primary prevention with statins when an alternate assumption is used: that the overall average statin effectiveness estimate in the CTT meta‐analysis (relative risk, 0.75 per 1 mmol/L reduction in LDL from statins for primary prevention) applies to all persons.
