# Supplementary material for: Population Impact & Efficiency of Benefit‐Targeted Versus Risk‐Targeted Statin Prescribing for Primary Prevention of Cardiovascular Disease
Source: J Am Heart Assoc. 2017 Feb 10;6(2):e004316. doi: 10.1161/JAHA.116.004316 (PMC5523747; doi:10.1161/JAHA.116.004316)
Supplement: Supplementary file 1 — Data S1. Two snippets of the PIE Model configuration code used to define Figure 2A and Figure S1. Table S1. Characteristics of NHANES 2011–2012 Participants Aged ≥20 Years Using Sample Weights, With and Without Multiple Imputation Table S2. Assumed Effects of Different Statins on Total, Low‐Density Lipoprotein (LDL), and High‐Density Lipoprotein (HDL) Cholesterol Figure S1. Prevention impact and efficiency for moderate‐intensity and high‐intensity statins with and without expanded eligibility. Impact (relative % reduction in ASCVD events) and number‐needed‐to‐treat (NNT) over 10 years to prevent each ASCVD event (lower NNT is more efficient) are illustrated across treatment thresholds for moderate‐ and high‐intensity statins targeted by expected absolute risk reduction (benefit‐based prescribing) and either limited to persons age 40 to 75 years, LDL <190 mg/dL and without diabetes mellitus or previous cardiovascular disease (base‐case analysis); or with expanded eligibility accounting for treatment of all persons with diabetes mellitus and LDL ≥190 mg/dL, and treatment of all adults of any age meeting the given expected absolute risk reduction threshold. Dashed line indicates 10% impact. ASCVD indicates atherosclerotic cardiovascular disease; LDL, low‐density lipoprotein cholesterol. Figure S2. Prevention impact and efficiency for moderate‐intensity statins with and without baseline risk interaction assumption. Our base‐case analyses assume an interaction between baseline risk and statin effectiveness, as detected by the Cholesterol Treatment Trialists (CTT)1 and operationalized by Thansssoulis et al2 (see Methods). This figure demonstrates that impact and efficiency are both substantially less favorable for primary prevention with statins when an alternate assumption is used: that the overall average statin effectiveness estimate in the CTT meta‐analysis (relative risk, 0.75 per 1 mmol/L reduction in LDL from statins for primary prevention) applies to all persons. [file JAH3-6-e004316-s001.pdf]

SUPPLEMENTAL MATERIAL

Data S1. Two snippets of the PIE Model configuration code used to define Figure 2a and Figure S1

Intervention-Threshold pair labeling (for all Figures):

```
* Choose and label the intervention-threshold pairs you want to graph (you can list ALL
POSSIBILITIES here, and decide later how to choose and order)
local pair1 `""Mod Statins, by Baseline Risk""'
local pair2 `""Mod Statins, by Expected ARR""'
local pair3 `""Mod Statins, by Age""'
local pair4 `""Mod Statins, perfect prediction""'
local pair5 `""High Statins, by Baseline Risk""'
local pair6 `""High Statins, by Expected ARR""'
local pair7 `""High Statins, by Age""'
local pair8 `""High Statins, perfect prediction""'
local pair9 `""Mod Statins with expanded elibigility, by Baseline Risk""'
local pair10 `""Mod Statins with expanded elibigility, by Expected ARR""'
local pair11 `""Mod Statins with expanded elibigility, by Age""'
local pair12 `""Mod Statins with expanded elibigility, perfect prediction""'
local pair13 `""High Statins with expanded elibigility, by Baseline Risk""'
local pair14 `""High Statins with expanded elibigility, by Expected ARR""'
local pair15 `""High Statins with expanded elibigility, by Age""'
local pair16 `""High Statins with expanded elibigility, perfect prediction""'
local pair17 `""Mod Statins without interaction, by Baseline Risk""'
local pair18 `""Mod Statins without interaction, by Expected ARR""'
local pair19 `""Mod Statins without interaction, by Age""'
local pair20 `""Mod Statins without interaction, perfect prediction""'
local pair21 `""Mod Statins if LDL>75, by Baseline Risk""'
local pair22 `""Mod Statins if LDL>75, by Expected ARR""'
local pair23 `""Mod Statins if LDL>75, by Age""'
local pair24 `""Mod Statins if LDL>75, perfect prediction""'
local pair25 `""Mod Statins with expanded eligibility, by Expected ARR""'
local pair26 `""High Statins with expanded eligibility, by Expected ARR""'
```

Configuration code for Figure 2a

```
* Name the figure (no spaces allowed).
local figname "Figure2a"

* Choose NNT type: ave or max
local NNTtype "max"        // "max" or "ave"

* Name the events your model predicts; This will go into the axis label
local eventname "ASCVD"

* Referring to the pair numbers defined in the list above, choose which you want to show in this
figure
local pairs_included "3 1 2 6 4"                                   //


* For each threshold, specify where you want a threshold symbol marker.  Each will have an entry in
the legend if that threshold is chosen for display.
* Note: You don't need to specify the Treat All thresholds, nor the thresh0 markers.
* Note2: The symbol won't actually show up on the graph unless it is included in the
Dataset3_Results.dta dataset.  If you find you want a marker somewhere that isn't included in
Dataset3_Results.dta, adjust your Step3.do file so that result is generated.
local thresh1_label "Treat if Baseline Risk>="
local thresh1_symbols "5 7.5"
local thresh1_max = 20

local thresh2_label "Treat if Expected ARR-mod>="
local thresh2_symbols "2.3"
local thresh2_max = 5

local thresh3_label "Treat if Expected ARR-high>="
```

```
local thresh3_symbols "1.5 2.0 2.3"
local thresh3_max = 5

local thresh4_label "Treat if Age>="
local thresh4_symbols "50 60"
local thresh4_max = 70
```

Configuration code for Figure S1

```
* Name the figure (no spaces allowed).
local figname "Figure_App1"

* Choose NNT type: ave or max
local NNTtype "ave"        // "max" or "ave"

* Name the events your model predicts; This will go into the axis label
local eventname "ASCVD"

* Referring to the pair numbers defined in the list above, choose which you want to show in this
figure
local pairs_included "2 6 25 26"



* For each threshold, specify where you want a threshold symbol marker.  Each will have an entry in
the legend if that threshold is chosen for display.
* Note: You don't need to specify the Treat All thresholds, nor the thresh0 markers.
* Note2: The symbol won't actually show up on the graph unless it is included in the
Dataset3_Results.dta dataset.  If you find you want a marker somewhere that isn't included in
Dataset3_Results.dta, adjust your Step3.do file so that result is generated.
local thresh2_label "Treat if Expected ARR-mod>="
local thresh2_symbols "2.3"
local thresh2_max = 5

local thresh3_label "Treat if Expected ARR-high>="
local thresh3_symbols "2.3"
local thresh3_max = 5
```

Table S1. Characteristics of NHANES 2011-2012 participants age≥20 years using sample weights, with and without multiple imputation

Characteristic	NHANES participants without missing risk factors (N= 2290)	NHANES participants with one or more missing risk factors* (N= 337)	p-value¶	US population estimates (using multiple imputation) (N=2627/sample‡)
Age, mean years ± SD	47 ± 17	48 ± 15	.62	47 ± 17
Sex, % male	48%	41%	.12	48%
Systolic blood pressure*, mean mmHg ± SD	121 ± 17	128 ± 18	.023	121 ± 17
Total cholesterol, mean mg/dl ± SD	193 ± 40	212 ± 47	.002	194 ± 41
LDL cholesterol, current†, mean mg/dl ± SD	115 ± 35	121 ± 36	.17	116 ± 35
HDL cholesterol, mean mg/dl ± SD	54 ± 15	48 ± 17	.001	53 ± 15
Smoking, % current	20%	16%	.12	20%
Diabetes, %	12%	23%	.001	15%
Current blood pressure medication use, %	29%	37%	.10	30%
Current statin use, %			.040	
- None	81%	86%		81%

- Standard dose¥	13%	6%		13%
- High intensity¥	6%	8%		6%
Prevalent ASCVD, %	8%	12%	.19	8%
10-year ASCVD Risk†, median % (interquartile range)	2.4% (0.5% - 9.7%)	---*	---*	2.5% (0.6% - 10%)
Eligible for targeted primary prevention with statins€, %	35%	---*	---*	34%
Expected ARR from Moderate-Intensity Statins among US adults eligible for targeted primary prevention with statins€, median % (interquartile range)	1.5% (0.7%-3.1%)	---*	---*	1.5% (0.7%-3.1%)

* - These participants are missing either systolic blood pressure (n=122), total cholesterol (n=187), LDL cholesterol (n=234), HDL cholesterol (n=187), smoking status (n=4) or diabetes (n=1), and as such we cannot estimate 10-year ASCVD risk, eligibility for statins, or Expected ARR. Estimates provided for each risk factor exclude participants missing these values. Two participants with missing systolic blood pressure were included in the first column because they had cardiovascular disease and therefore did not require a systolic blood pressure measurement for calculation of ASCVD risk[3, 4]. Note that 2.6% of all measurements are imputed in the imputed datasets (= (122+187+234+187+4+1+2) / (2627 persons*11 measurements)).

¶ - p-values for comparison of participants with and without any missing values are calculated for each characteristic using sample weights multi-stage sampling design variables, and include only those participants who are not missing that characteristic*.

‡ - Number of observations in each of the 10 imputed datasets.

† - Atherosclerotic cardiovascular disease (ASCVD) risk was estimated using the algorithm described in the 2013 ACC/AHA Guideline on the Assessment of Cardiovascular Risk[5] for persons without pre-existing cardiovascular disease, or an alternate Framingham-based risk equation[3, 4], with extrapolation to 10 years for persons with and without pre-existing cardiovascular disease, respectively (see Methods). Expected ARR for Moderate-Intensity Statin therapy was estimated using the formulation developed by Thanassoulis et al[2], see Methods.

¥ - We assumed persons reporting use of Pravastatin, Lovastatin, Simvastatin or Fluvastatin were using “Standard dose” statins and persons using Atorvastatin, Rosuvastatin or Pitavastatin were using “High dose” statins.

€ - NHANES participants age 40-75 years with LDL<190 mg/dl, no diabetes, no prior cardiovascular disease, and not already on a statin are eligible for targeted primary prevention with statin therapy.

NHANES – National Health and Nutrition Examination Survey; SD – Standard deviation; LDL – Low-density lipoprotein; HDL – High-density lipoprotein; Statin – HMG Co-A Reductase Inhibitor; ARR – Absolute risk reduction

Table S2. Assumed effects of different statins on total, low-density lipoprotein (LDL) and high-density lipoprotein (HDL) cholesterol

Statin	Number of NHANES participants using the indicated statin	Assumed effects* of the indicated statin on:		
		Total cholesterol	LDL cholesterol	HDL cholesterol
Pravastatin	60	-27%	-34%	+5%
Lovastatin	41	-27%	-34%	+5%
Simvastatin	244	-27%	-34%	+5%
Fluvastatin	0	-27%	-34%	+5%
Atorvastatin	105	-37%	-48%	+5%
Rosuvastatin	50	-37%	-48%	+5%
Pitavastatin	2	-37%	-48%	+5%
More than one statin	0	N/A	N/A	N/A
None of the above†	2125	---	---	---

* - For persons reporting use the indicated statin, we assumed the statin caused reductions in total and LDL cholesterol and increases in HDL as above (using Maron et al 2000; see Reference 8 in the main article), and used these assumptions to calculate pretreatment values for the purpose of risk estimation (see Methods).

† - Total N for this analysis is the 2627 NHANES participants in our target population (see Methods).

NHANES – National Health and Nutrition Examination Survey; LDL – Low-density lipoprotein; HDL – High-density lipoprotein

Supplemental Figure Legends:

Figure S1. Prevention Impact and Efficiency for Moderate-Intensity and High-Intensity Statins with and without expanded eligibility. Impact (relative % reduction in ASCVD events) and number-needed-to-treat (NNT) over 10 years to prevent each ASCVD event (lower NNT is more efficient) are illustrated across treatment thresholds for moderate and high intensity statins targeted by expected absolute risk reduction (benefit-based prescribing) and either limited to persons age 40-75 years, LDL<190 mg/dl and without diabetes or prior cardiovascular disease (base-case analysis); or with expanded eligibility accounting for treatment of all persons with diabetes and LDL≥190 mg/dl, and treatment of all adults of any age meeting the given expected absolute risk reduction threshold. Dashed line indicates 10% impact. ASCVD – Atherosclerotic cardiovascular disease; LDL – Low-density lipoprotein cholesterol

Figure S2. Prevention Impact and Efficiency for Moderate-Intensity Statins with and without baseline risk interaction assumption. Our base case analyses assume an interaction between Baseline Risk and statin effectiveness, as detected by the Cholesterol Treatment Trialists (CTT)[1] and operationalized by Thansssoulis et al[2] (see Methods). This Figure demonstrates that impact and efficiency are both substantially less favorable for primary prevention with statins when an alternate assumption is used: that the overall average statin effectiveness estimate in the CTT meta-analysis (relative risk 0.75 per 1 mmol/L reduction in LDL from statins for primary prevention) applies to all persons.

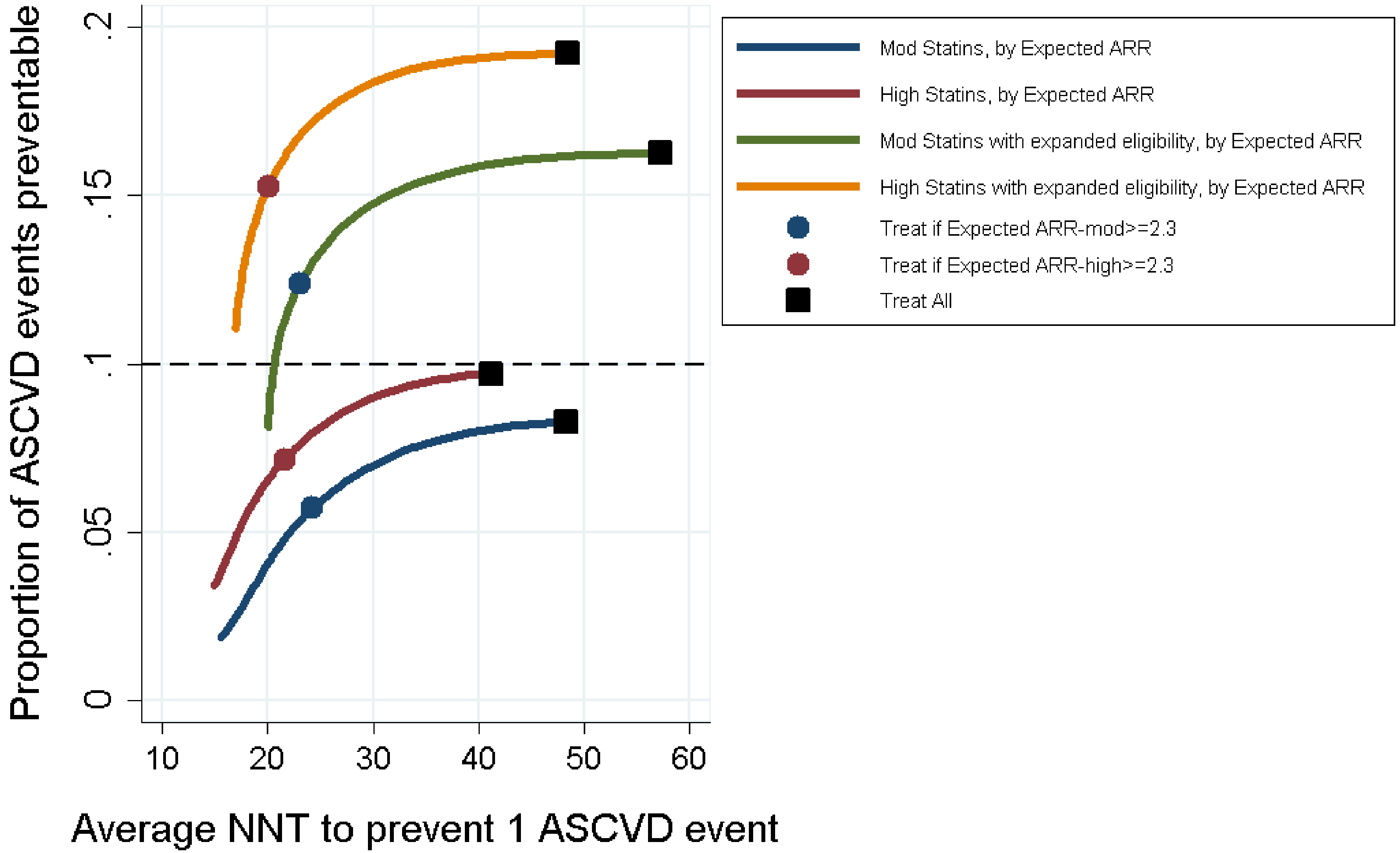

Proportion of ASCVD events preventable
0
.05
.1
.15
.2
10
20
30
40
50
60
Average NNT to prevent 1 ASCVD event
Mod Statins, by Expected ARR
High Statins, by Expected ARR
Mod Statins with expanded eligibility, by Expected ARR
High Statins with expanded eligibility, by Expected ARR
Treat if Expected ARR-mod>=2.3
Treat if Expected ARR-high>=2.3
Treat All

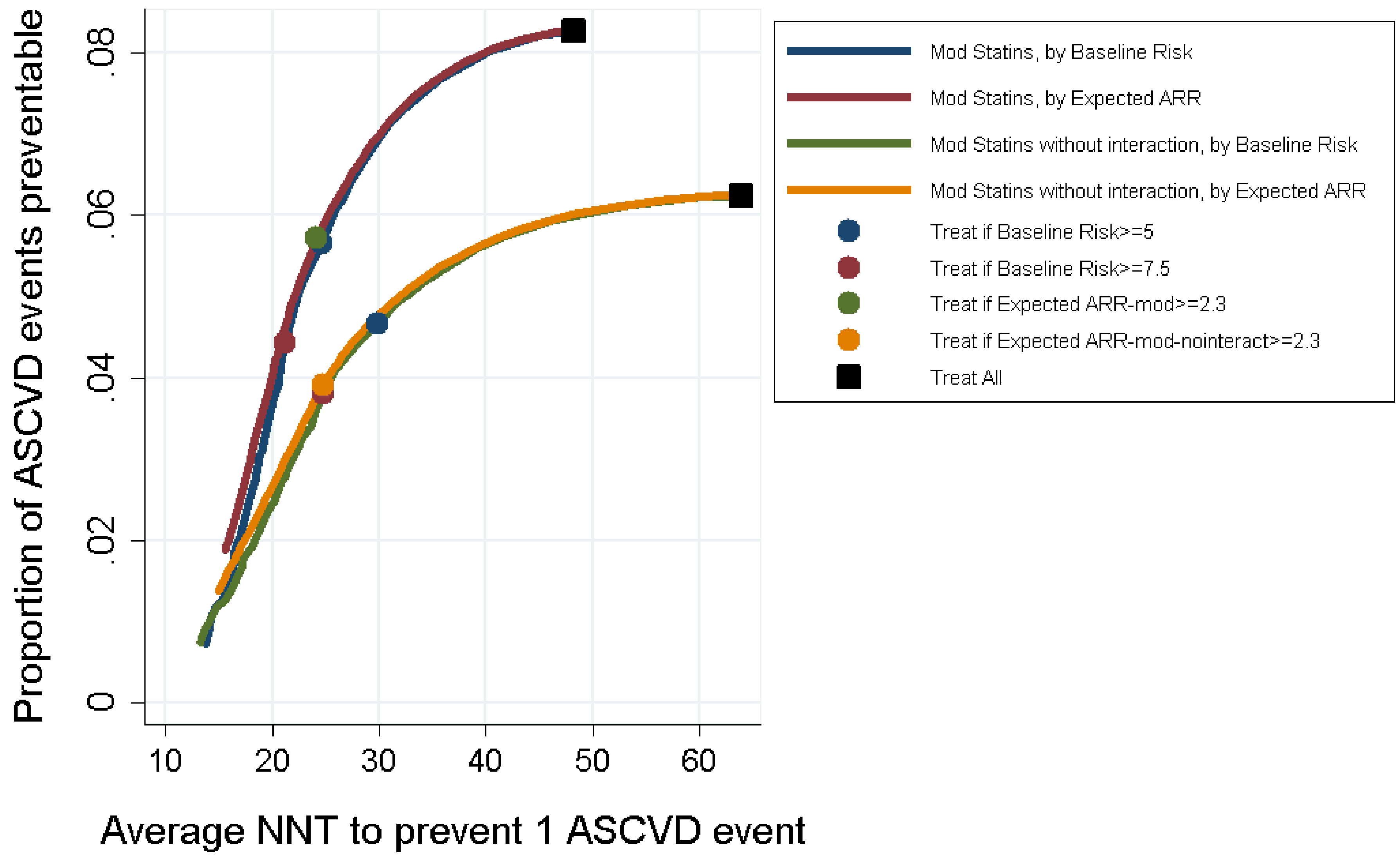

Proportion of ASCVD events preventable
0
.02
.04
.06
.08
10
20
30
40
50
60
Average NNT to prevent 1 ASCVD event
Mod Statins, by Baseline Risk
Mod Statins, by Expected ARR
Mod Statins without interaction, by Baseline Risk
Mod Statins without interaction, by Expected ARR
Treat if Baseline Risk>=5
Treat if Baseline Risk>=7.5
Treat if Expected ARR-mod>=2.3
Treat if Expected ARR-mod-nointeract>=2.3
Treat All

Supplemental References:

1. Mihaylova B, Emberson J, Blackwell L, Keech A, Simes J, Barnes EH, Voysey M, Gray A, Collins R, Baigent C. The effects of lowering ldl cholesterol with statin therapy in people at low risk of vascular disease: Meta-analysis of individual data from 27 randomised trials. *Lancet*. 2012;380:581-590
2. Thanassoulis G, Williams K, Kimler Altobelli K, Pencina MJ, Cannon CP, Sniderman AD. Individualized statin benefit for determining statin eligibility in the primary prevention of cardiovascular disease. *Circulation*. 2016;133:1574-81. doi: 10.1161/CIRCULATIONAHA.115.018383. Epub 2016 Mar 4.
3. Recurrent coronary heart disease. Framingham Heart Study. Accessed at https://www.framinghamheartstudy.org/risk-functions/coronary-heart-disease/recurrent.php on Nov 29, 2016.
4. D'Agostino RB, Russell MW, Huse DM, Ellison RC, Silbershatz H, Wilson PW, Hartz SC. Primary and subsequent coronary risk appraisal: New results from the framingham study. *Am Heart J*. 2000;139:272-281
5. Goff DC, Jr., Lloyd-Jones DM, Bennett G, Coady S, D'Agostino RB, Sr., Gibbons R, Greenland P, Lackland DT, Levy D, O'Donnell CJ, Robinson J, Schwartz JS, Shero ST, Smith SC, Jr., Sorlie P, Stone NJ, Wilson PW. 2013 acc/aha guideline on the assessment of cardiovascular risk: A report of the american college of cardiology/american heart association task force on practice guidelines. *Circulation*. 2014;129(25 Suppl 2):S74-5.
